# Farm-Level Effects of Emissions Tax and Adjustable Drainage on Peatlands

**DOI:** 10.1007/s00267-021-01543-1

**Published:** 2021-10-14

**Authors:** Tuomo Purola, Heikki Lehtonen

**Affiliations:** Luke Natural Resources Institute Finland - Economics and society, Latokartanonkaari 9, FI-00790 Helsinki, Finland

**Keywords:** Climate-change mitigation, Agriculture, Land use, Farm management, Farm income, Climate policy

## Abstract

Drained agricultural peatlands emit significantly higher amounts of greenhouse gas (GHG) emissions per hectare than mineral soils. GHG abatement costs for representative cereals (CF) and dairy (DF) farms in southwestern Finland were estimated by integrating an emission-based tax together with an option to invest in a subsidized adjustable drainage system on peat soils in a farm-level dynamic optimization model. With an average 10% share of peat soils from overall farm area, emissions tax rates over 15 (CF) and 19 (DF) €/tCO_2_e triggered adjustable drainage investments with a significant reduction in GHG emissions per ha, when assuming no crop-yield effect from the adjustable drainage. Abatement costs for emissions tax rates €12–50/tCO_2_e/ha were €16–44/tCO_2_e (CF) and €26–51/tCO_2_e (DF) for whole farm-soil emissions, depending on the share of peatlands on the farm, on the yield effects of adjustable drainage, and on crop prices. High emissions tax rates imply higher abatement costs since farms have a limited capability to adjust their production and land use. Thus, emissions reductions from peatlands can be achieved at reasonable costs when investing in adjustable drainage on peatlands. The income losses due to emissions tax, however, are high, but they can be compensated for farmers by lumpsum payments independent of their production decisions. Since existing agricultural policies such as the EU CAP system may have limited effectiveness on GHG emissions, the emissions tax and adjustable drainage on peatlands could promote GHG abatement significantly on farms and areas with abundant peatlands.

## Introduction

In Finland, agricultural production and its land use produce approximately 16 Mt CO_2_e/year greenhouse gas (GHG) emissions, which account 20% of total emissions (Statistics Finland 2021a). Reducing agricultural GHG emissions from current levels is important for meeting a national target of climate neutrality by 2035 (Koljonen et al. 2020). Reducing GHG emissions from agriculture significantly is however a challenge. Recent studies show that one potential source of emissions where reductions could be made effectively is peatlands. Peatlands comprise only 11% of agricultural land, but they produce over 50% of the total GHG emissions from agriculture and 75% of the emissions from agricultural land use in Finland (Kekkonen et al. 2019; Koljonen et al. 2020; Lehtonen et al. 2020).

The potential of peatlands in GHG emissions mitigation has also gained attention globally. Peatlands cover about 3% of the land area, store 30% of the soil carbon, and produce 6% of all carbon dioxide (CO_2_) emissions globally (Joosten et al. 2012). Peatlands provide ecosystem services such as water provision, nutrient cycling, food and fiber production, recreation, habitats, and biodiversity, and are of interest to countries that aim for significant GHG emissions reductions (Bonn et al. 2016). For example, the UK GHG National Inventory has recently included emissions from degraded peatlands in to the inventory (UK Government 2021). This would lead to higher GHG emissions in the baseline but would also provide more opportunities for emissions reductions, for example, through peatland restoration.

Soil carbon has significant value for society as a means of carbon sequestration. Costs and benefits of different peatland restoration and conservation practices, which aim to maintain soil organic carbon, have been studied in different regions in Europe. Graves and Morris (2013) estimated degradation on peatlands and the associated loss of soil carbon under different land-use and climate-change scenarios in United Kingdom in the period 2012–2080. When valuing emissions cost at £57/tCO_2_e, the CO_2_ sequestration benefit of peat restoration was estimated to about £300/ha for 2012 and about £40/ha for peatland conservation on extensive grassland. Moxey and Moran (2014) estimated the abatement costs of achieving emissions reductions targets and used this as a value for carbon benefits. They valued the capital costs of restoration to range from £200/ha–£10,000/ha and ongoing costs in the range of £25/ha–£400/ha, depending on the circumstances.

Röder and Osterburg (2012) estimated that rewetting peatlands in Germany would result in GHG mitigation costs in the range of €10–45/tCO_2_e without engineering or transaction costs.

Krimly et al. (2016) calculated the GHG abatement costs from peatlands in Germany. According to their results, the conversion of arable land of peat-soil type into medium-drained intensive grassland would lead to high abatement costs up to €92/tCO_2_e, while the abatement costs of the rewetting and conversion as wet grassland would range from 5 to 57 €/tCO_2_e.

Grossmann and Dietrich (2012) calculated abatement cost estimates for cases of changed peatland management in a context of agricultural peatlands in the Elbe river basin in North-East Germany. They reported abatement costs as low as €7–14/tCO_2_e for peatland restoration, while the median-estimated abatement costs for certain peatland-stabilization scenarios were within a range of €10–20/tCO_2_e.

Rewetting, i.e., raising the level of the watertable on peatlands, is seen as an effective way of reducing GHG emissions in agriculture in northern conditions (Kekkonen et al. 2019). Such emissions reductions could be also accounted for in national GHG inventory, which is fully consistent with the United Nations Framework Convention on Climate Change (UNFCCC), the Kyoto Protocol, and the EU greenhouse gas monitoring mechanism that obliges members to monitor and report greenhouse gas emissions on an annual basis. A recent climate roadmap of the unions of Finnish agricultural producers (Lehtonen et al. 2020) find and suggest that the least productive peatlands could be rewetted and significant reductions of GHG emissions could be achieved. However, raising the watertable close to the surface is problematic for farmers because it restricts both the choice of cultivated crops and the machinery use of farms since conventional agricultural machinery is not feasible on very wet and soft peatlands (Wichtmann et al. 2016). Furthermore, there are limited markets and insufficient demand for crops, which would be feasible for paludiculture (wet agriculture and forestry on peatlands) (Niemi 2020) in Finland, as well as in other European countries. Thus, as the opportunity cost of adopting new sustainable practices is high, countries are oriented toward producing conventional crops and livestock products on drained agricultural land (Ferré et al. 2019). The share of peatland soils is highly variable in Finland (Kekkonen et al. 2019) and many farms and agriculture in smaller regions in Finland are quite dependent on using drained peatlands for agricultural production. Still, significant reductions in agricultural GHG emissions is not possible without reducing emissions from peatlands. Hence, there is an increasing demand for solutions that might facilitate both significant reductions in GHG emissions and still allow conventional agricultural production. The aim of this study is to analyze to what extent and cost GHG emissions from Finnish peatlands could be mitigated while still keeping the existing fields of peat soil in agricultural use.

Adjustable subsurface drainage is a potential solution that simultaneously enables conventional agricultural production and reduces emissions from peatlands. The watertable can be kept at a higher level, for example 30 cm below the surface or higher, temporarily or most of the year, while the watertable could be decreased for example to 60 cm below the surface to allow machinery operations and farm work when necessary during the sowing and harvesting periods. The oxygenated soil layer is thinner when the watertable is high, thus, the decomposition of organic matter is reduced, and there will be fewer greenhouse gas emissions into the atmosphere. The watertable is usually regulated by control wells with a simple mechanism for adjusting the discharge pipe height (Regina et al. 1996, 2015; Flessa et al. 1998; Myllys 2019).

For an individual farmer, it is important to know the costs of adjustable drainage and its effects on crop yields, while society as a whole is interested in GHG-mitigation costs. Some estimations of the effects of adjustable drainage on GHG emissions and crop yields have been made. A study by Evans et al. (2021) suggests that adjusting the watertable depth is the most efficient way of controlling greenhouse gas emissions from peatlands in the UK context. Every 10 centimeters of reduction in the water-table depth was estimated to reduce the net warming impact of emissions by 3 tons of CO_2_e per hectare per year, until the water-table depth is less than 30 centimeters and emissions reductions continue until the depth is within 10 centimeters of the surface.

Myllys (2019) studied the effects of the water-table level on cereal yields and GHG emissions on Finnish peatlands with adjustable drainage in the years 2014–2016. A 10-cm rise in the water table reduced the GHG emissions by 20%. Yields were reduced slightly on the average. When there was high precipitation during the growing season, yields were reduced by about 1000 kg/ha, whereas 1000 kg/ha yield gain was obtained when the precipitation was low.

A study by Matysek et al. (2021) found that raising the watertable from −50 cm to −40 and −30 cm on East Anglian fenlands (UK) reduced CO_2_ emissions, while CH_4_ emissions were not significantly affected. Simultaneously, the yields of romaine lettuce were reduced significantly. A water-table level of −40 cm was seen as a compromise where emissions are cut but agricultural production is maintained.

A study by Weideveld et al. (2021) evaluated the effects of subsoil-irrigation systems on GHG emissions (CO_2_, CH_4_, and N_2_O) for four dairy farms on drained peat meadows in the Netherlands in 2017–2018. The system prevented the groundwater table dropping below −60 cm. The outcome of the study was that the subsoil-irrigation system did not reduce the GHG emissions from peat meadows.

While some literature suggests varying evidence concerning the effectiveness of adjusting the watertable in reducing GHG emissions in different countries in Europe, based on larger literature study, Kekkonen et al. (2019) concluded that raising the watertable would decrease GHG emissions from peatlands in northern conditions, e.g., in Finland. The IPCC (2014) has estimated GHG emissions for different land-use options for different soil types (Table 1 in 2.3.2). These coefficients, which are also used in national greenhouse gas inventories (Statistics Finland 2021a) include both CO_2_ and N_2_O emissions from agricultural land converted as CO_2_ equivalents.

**Table 1:** Greenhouse gas emissions (N_2_O and CO_2_) from mineral and organic soils for different land use options converted as tCO_2_e/ha (IPCC 2014)

	GHG emissions tCO_2_e/ha		
Land use option	Mineral soil	Organic soil	Adjustable drainage in organic soil
Spring wheat	2.0	35.1	21
Winter wheat	2.0	35.1	21
Feed barley	2.0	35.1	21
Malting barley	2.0	35.1	21
Oats	2.0	35.1	21
Oilseed rape	2.0	35.1	21
Grass	1.0	25.3	14.9
Set-aside	0	25.3	14.9
NMF	0	25.3	14.9

Current agricultural policy is poor at incentivizing farmers to maintain a high watertable on agricultural land, while there are incentives for keeping perennial crops on peatlands. There are two main reasons for this. First, a high watertable restricts the crop choice significantly since not all crops tolerate a high watertable and those that do are not all eligible for CAP subsidies. In fact, agricultural-support payments, including CAP-decoupled payments, payments for less-favored areas (LFAs), and agri-environmental payments, including payments for nature-management fields (NMFs) (a specific type of grassland aimed for nature conservation), require sufficient drainage on fields. Thus, it is less risky and less costly for a farmer, at least in the short term, not to invest in dams and other means of raising the water table in farmlands but rather to keep the watertable low and thus receive the farm subsidies mentioned above.

Second, water protection and biodiversity maintenance, not GHG emissions reduction, have been the focus areas of the agri-environment scheme implemented under the EU Common Agricultural Policy (CAP) in Finland (Hyvönen et al. 2020). This scheme pays subsidies for certain farm-level practices based on farm-level costs and foregone income, but does not reward farmers for GHG emissions reduction directly. The scheme includes only a few measures for peatland protection or maintenance of soil carbon on agricultural peatlands. Nature-management fields (NMF) with perennial grasses are incentivized with specific-area payments and this applies also to peatlands. Long-term perennial grass has been encouraged by a scheme where a farmer commits to no-tillage and continuous perennial grass on peatlands for 20 years. However, the payment based on cost compensation is only approximately €100/ha, and thus, this scheme has been adopted by only a few farmers and the budget allocation of public funds has been small for this measure (Hyvönen et al. 2020). Investment subsidies for adjustable drainage cover 40% of the investment costs (Pro Agria 2019). Despite this, adjustable drainage has been so far installed on only approximately to 3000 hectares of peatlands in Finland and there is no obligation, incentive, or monitoring to keep the watertable high on the peatlands (Hyvönen et al. 2020).

The weakness of the CAP system at mitigating GHG emissions in agriculture has been found also in other studies and elsewhere in Europe. Stronger programmed action has been suggested to meet local environmental and climate-related needs (European Court of Auditors 2017). Recent proposals for the CAP for the period 2022–2027 see a high need for mitigating climate change and there are increasing ambitions to reduce GHG emissions from agriculture (European Commission 2020).

To sum up, a farmer is very likely to lose money by permanently rewetting peatlands since there will be less crop income, fewer farm subsidies, and higher costs compared with the conventional mainstream practice where the water table is low (Ferré et al. 2019; Miettinen et al. 2020). Since the current agricultural policy system is obviously weak at addressing the challenge of achieving major reductions in GHG emissions, it is necessary to evaluate what could be achieved with policy measures incentivizing GHG emissions reductions directly. While many policy measures and incentives have been used with the aim of reducing GHG emissions from peatlands in many countries in Europe (Wichmann 2018), emissions taxes on GHG emissions are rare or nonexistent in Europe. However, the EU is considering a carbon tax as a measure for taxing imports and their GHG emissions in some sectors (not agriculture) under the Carbon Border Adjustment Measure (European Commission 2021). Emissions taxes on GHG emissions from soils are one way of internalizing the public cost of GHG emissions, not otherwise considered in farmers’ decision-making. For this reason, economists traditionally like such taxes. Hence, a tax on soil emissions as an emissions-reduction incentive is analyzed in this paper.

In this article, the effects of different GHG emissions tax rates on GHG emissions, production and income are analyzed for the two most important and typical farm types in Finland: a cereals farm and a dairy farm. These farm types are common, especially in regions with abundant peatlands (Official Statistics of Finland (OSF) 2021). Simulations show how a large tax will trigger investments in adjustable drainage and what other farm-level changes would follow from a GHG emissions tax. The results are based on a dynamic-optimization farm model with a time span of 30 years. The model is capable of accounting for explicit crop-rotation dynamics with crop-yield effects on different field parcels. At this point, methane emissions from livestock, which are approximately 15% of the total agricultural GHG emissions in Finland (Lehtonen et al. 2020; Statistics Finland 2021b) are not considered. This is because research results on the effects of methane inhibitors suggest high variability and high costs for reducing methane emissions from dairy cows in Finnish conditions (Sairanen 2021). Hence, methane-emission abatement costs are very likely to be high and we do not consider them in this study, which focuses on GHG emissions from soils.

The aim of this paper is to evaluate the GHG emissions abatement costs from soils on farms with peatlands, and how a policy instrument addressing GHG emissions directly such as a GHG emissions tax could affect GHG emissions, farm production, and farm income.

This analysis is relevant and valid in the conditions in Finland and other Nordic countries such as Sweden and Norway located in Northern Europe. There are also several other countries in Europe with abundant peatlands: Estonia, Germany, the Netherlands, the United Kingdom, and Ireland (Tanneberger et al. 2017). Thus, the results might be of interest to other countries as well since the EU has confirmed a target of climate neutrality by 2050 (European Commission 2018).

## Materials and Methods

### Study Region

In this study, an analysis was implemented for the Varsinais–Suomi province, which is located in southwest Finland and is one of the most favorable agricultural regions in Finland. The average length of the thermal growing season was 180–200 days in 1981–2010. The effective temperature sum was 1300–1450 degrees, and the average precipitation in the growing season was 350–400 mm (Pirinen et al. 2012). The growing season usually starts in the last week of April and ends at the end of October. About 56% of farms are cereal farms in this region. The share of dairy farms is only 3% of total farms in the region and only 2.5% from all dairy farms in Finland. Dairy farms in the region have on average 52 cows and they produce 2.8% of the total milk production in Finland (Luke 2020). The average size of all dairy farms in Finland was 44 cows and average size of all farm types 61 ha in 2020 (Luke 2021). Dairy milk production is primarily based on grass forage-based feeds, harvested as silage and stored to be used during a long 8–9-month period of in-house feeding when there is no option for pasturing due to climate conditions. Cereals and protein supplements, especially crushed oilseed, are important in dairy cow feeding as well, but most of the feed intake in terms of dry matter comes from forage grass silage (Huhtamäki 2021).

Most farms in the region apply short crop rotations or monocropping (Vuorio et al. 2006). Varsinais–Suomi farms are affected by the 5% minimum area requirement for an ecological area under the EU’s CAP (set aside is accepted as an ecological area), and by the maximum area restriction (15%) for nature-management fields (NMF) and maximum overall set-aside area (set-aside and NMF) restrictions (25%), as specified in the CAP agrienvironmental scheme implemented at the national level (Niemi and Väre 2017). The area of cultivated organic soils is only 1.7% of the total cultivated area in the region, but this can be substantially higher in certain smaller areas within the region (Kekkonen et al. 2019).

### DEMCROP Model

A dynamic economic model of farm management and crop rotation (DEMCROP) was utilized in the analysis. The DEMCROP model has been applied earlier in studies by Lehtonen et al. (2014, 2016a) and Liu et al. (2016) and most recently by Purola et al. (2018) and Purola and Lehtonen (2020). The DEMCROP model utilizes dynamic optimization and thus can handle dynamic intertemporal decisions together with short-term decisions. DEMCROP maximizes the discounted expected future gross margin subtracted by a weighted covariance of the gross margin taking into account that risk-averse farmers make trade-offs between expected profit and variance on profit. The model solves optimal parcel-specific land allocation and input-use problems in given scenarios, which can include different input and output prices, policy environments, and time spans, taking into account crop rotation and other dynamics, both in the short and long term.

DEMCROP can be formulated and solved by nonlinear programming as follows:

$$\begin{array}{l}{{{\rm{max}}}}\mathop{\sum }\limits_{t = 1}^T \mathop {\sum }\limits_{p = 1}^P \mathop {\sum }\limits_{l = 1}^L \frac{1}{{\left( {1 + r} \right)^t}}\left( Y\left( {A\left( {p,t,l} \right),p,t,l} \right)\left( {A\left( {p,t,l} \right)} \right.P\left( l \right)\right.\\ \quad \left. +\, S\left( l \right) - C\left( {p,t,l} \right)\right)- \theta \mathop {\sum }\limits_{t = 1}^T \mathop {\sum }\limits_c \mathop {\sum }\limits_{c2} \frac{1}{{\left( {1 + r} \right)^t}}A^{\prime}XA\end{array}$$

subject to

$$\mathop {\sum }\limits_{\forall i} A\left( {p,t,l} \right) \le 1$$

where *A*(*p,t,l*) is the area allocation for crop *i* at time (year) *t* on field parcel *p*. *Y*( ∙ ) is the crop yield level, dependent on the nitrogen-fertilization level (which, in turn, depends on expected crop and fertilizer prices), and past area allocations on the field parcel *p* (there are yield losses due to monocultural cultivation). *P*(*i*) is the expected average market price of crop *i*. *S*(*i*) is the subsidy paid per hectare, and *C*( ∙ ) is cost per hectare. *X* is the covariance matrix of crop-specific gross margins calculated based on past crop yields and the prices of the inputs and outputs. This means that past covariation of the gross margins is assumed, while the expected average market prices of the crops are fixed and outside the control of the farmer. The model is presented in more detail by Purola et al. (2018).

The main features of the model can be summarized as follows. A farm has a certain amount of agricultural land that is divided into soil parcels. The soil types of the parcels include mineral soils and peat soils, which release different levels of GHG emissions, which may however be influenced by crop choice, perennials, or annual crops. The farm has a fixed number of land-use options (crops and set-asides).

Yield losses caused by monoculture cultivation are specified in a fixed-yield penalty matrix. Crop and field parcel-specific management decisions include liming, fertilization, and fungicide-use decisions. The soil pH, affected by liming decisions, affects the crop yields in each field parcel. Nitrogen fertilization is crop specific and is optimized based on crop and fertilizer prices. Mineral nitrogen fertilization is assumed to reduce the soil pH.

Liming is a dynamic decision since liming can raise the soil pH to an optimal level immediately, but it will take several years before the soil pH decreases to low levels again. In the model, the pH value of the soils decreases annually by a certain amount due to natural factors and also due to the level of inorganic fertilization in the soil parcel.

Fungicide treatment is determined based on annual input and output prices and does not have dynamic consequences. Logistics costs related to the cultivation of each field parcel depend on crop and management choices and on the distance between the parcel and the farm center. Logistics costs include driving times, fuel consumption, and labor needs.

The model structure does not imply large changes in the production intensities of single crops between the field parcels, but the nitrogen fertilization and crop yields are different between crops. Hence, the crop choice drives the intensity of production in the field parcels. The nitrogen fertilization is zero for NMF and set-aside plots, close to 100 kg N/ha for cereals, and approximately 170 kg N/ha for forage grass.

### Input Data

#### Historical yield and price data

Historical data comprise 10 years from period 2010–2019 for crop yields, variable costs, subsidies, and output price data. Average crop yields in the period are extracted from official farm statistics (OFS, 2018) for the Varsinais–Suomi region in Finland (Appendix Table A1). The average variable costs of the crops are derived from a recent version of a dynamic regional sector model of Finnish agriculture (DREMFIA) (Lehtonen 2001; Lehtonen and Niemi 2018), which relies on annually validated input prices and approximations of the average use of inputs per crop in each region. The use of production inputs per ha available gross-margin calculations provided by Pro Agria (2021) are close to the ones we use in this study. Subsidies are calculated by assuming that a farmer receives all available basic subsidies. In the case of agri-environmental subsidy, only basic measures available for all farmers are assumed. Output prices are average crop prices in the period. In the case of a dairy farm, only crop production is modeled. It is assumed that the dairy farm obtains a certain value from grass forage per ton and it has to produce at least a certain minimum amount of grass feed for the cattle.

The Internet-based “Paalilaskuri” (grass bale counter) provided by (MTT 2021) was used to determine the price of grass. The price is based on the feed-energy content by using feed barley as a reference feed and assuming a 40% dry matter content of grass-forage feed.

#### GHG-emission coefficients

Greenhouse gas emissions for different land-use options for different soil types (Table 1) are based on coefficients estimated by IPCC (2014). These coefficients, which are also used for the national greenhouse gas inventory (Statistics Finland 2021a), include both CO2 and N2O emissions from agricultural land calculated as CO2 equivalents. It is notable that GHG emissions from livestock, such as methane from ruminants or nitrous oxide from manure management, are not considered in this study since the methane-reduction costs are likely to be high (Sairanen 2021) and reducing N2O emissions from manure management is challenging (Regina et al. 2014).

### Model Validation

The model has been validated for the Varsinais–Suomi region in an earlier study by Purola and Lehtonen (2020). Base scenarios were simulated with updated input data (2010–2019) with the same risk-aversion parameter value (0.000002). This small risk-aversion parameter, used also in Purola & Lehtonen (2020), means low risk aversion and is compatible with the literature, which suggests that farmers in developed countries are typically relatively little risk-averse (Raskin and Cochran 1986) though some risk behavior can be observed. Nevertheless, risk aversion plays little role in this study.

We assume a farm has ten equally sized soil parcels. The distance between the farm center and soil parcels varies from 0 to 7 km, averaging at 3 km. Nine parcels are assumed to be mineral soil types and one parcel (parcel 6, 2 km from farm center) is assumed to be an organic soil type. On a cereals farm, there are eight land-use options, six different crops (spring wheat, winter wheat, feed barley, malting barley, oats, and oilseed rape) and two types of set-asides (normal set-aside and nature-management field). On a dairy farm, there is also a grass-production option, in addition to the options of the cereals farm. A minimum level of 5.5 is given for the soil pH on mineral parcels and 5.0 on organic soil parcels to prevent a very low soil pH and crop-yield levels since agricultural-policy conditions require the land to be kept in good agricultural condition (Lehtonen and Niemi 2018).

The base-scenario results for cereals and dairy farms are presented in Table 2A, B. The results for average yields, soil pH, nitrogen fertilization, and fungicide treatment correspond closely to the regional averages. On a cereal farm, all crops included in the model are cultivated. Oats is the main crop on a cereals farm, but also spring wheat, feed barley, and oilseed are cultivated regularly (Table 2A). Oilseed has a good gross margin, and it is also a good breaking crop for cereal-dominated rotations. However, due to its considerable yield loss due to plant-disease risks in monoculture cultivation, a few year breaks are needed between its cultivation on the same soil parcel. For distant parcels, the logistic costs are high and therefore nature management fields and feed crops, which require less management than higher-value crops, are allocated to them more often.

**Table 2:** A. Land use on a cereals farm (CF) with average prices without an emissions tax. B. Land use on a dairy farm (DF) with average prices without an emissions tax

	SWheat	WWheat	FBarley	MBarley	Oats	Oilseed	Set-aside	NMF
PARCEL 1	7%	7%	7%	27%	33%	15%	0%	5%
PARCEL 2	20%	0%	3%	27%	27%	18%	0%	5%
PARCEL 3	12%	7%	23%	10%	30%	13%	0%	5%
PARCEL 4	10%	3%	20%	17%	33%	13%	0%	3%
PARCEL 5	7%	0%	35%	0%	25%	13%	0%	20%
PARCEL 6	50%	10%	0%	0%	23%	17%	0%	0%
PARCEL 7	28%	0%	17%	0%	25%	13%	0%	17%
PARCEL 8	40%	0%	7%	0%	22%	13%	0%	18%
PARCEL 9	18%	0%	23%	0%	17%	13%	0%	28%
PARCEL 10	17%	0%	23%	0%	18%	13%	0%	28%
AVG.	20.8%	2.7%	15.8%	8.0%	25.3%	14.3%	0.0%	13.0%

	SWheat	WWheat	FBarley	MBarley	Oats	Oilseed	Grass	NMF
PARCEL 1	0%	0%	18%	0%	2%	10%	70%	0%
PARCEL 2	0%	0%	22%	0%	2%	10%	65%	0%
PARCEL 3	0%	0%	20%	0%	2%	7%	72%	0%
PARCEL 4	0%	0%	18%	0%	2%	10%	70%	0%
PARCEL 5	0%	0%	22%	0%	2%	7%	70%	0%
PARCEL 6	0%	2%	15%	0%	0%	10%	73%	0%
PARCEL 7	0%	0%	16%	0%	4%	7%	73%	0%
PARCEL 8	0%	0%	18%	0%	5%	7%	70%	0%
PARCEL 9	1%	0%	20%	0%	5%	0%	74%	0%
PARCEL 10	0%	0%	23%	0%	3%	0%	73%	0%
AVG.	0.1%	0.2%	19.3%	0.0%	2.6%	6.7%	71.0%	0.0%

The average pH value of the soils is close to the observed average (6.1) as liming activities and fungicides are used for 45% of the cereal area (Table 3). The yields are close to the average yields observed in the region during 2010–2019. The average gross margin is €110/ha. The average GHG emissions are 5.05 tCO_2_e/ha from whole farms and 35.1 tCO_2_e/ha from organic soil parcels. Annual crops are cultivated on organic soil parcels, which led to higher GHG emissions compared with set-asides. Total production calculated as feed energy is 34,681 GJ/ha, thus, the emissions per unit produced are 0.146 CO_2_e/GJ.

**Table 3:** Main results for the cereals (CF) and dairy farm (DF) with average data without emissions tax

Average crop prices	CF	DF
Value (€) of objective function over 30 years	33 083	63 252
Average CE gross margin €/ha/year	110	211
Average pH	6.11	6.50
Average GHG emissions tCO_2_e/ha	5.05	3.32
Average GHG emissions from parcel 6 tCO_2_e/ha	35.1	27.9
Total production, GJ/ha	34 681	35 136
GHG emissions tons CO_2_/GJ	0.146	0.094
Average yields, kg/ha		
Spring wheat	3874	4174
Winter wheat	4868	4577
Feed barley	3628	4167
Malting barley	3946	3979
Oats	3740	4023
Oilseed rape	1654	1749
Grass	–	11901
Percentage of fungicide treatment area (*SW, WW, FB, MB)	45%	43%

Most of the land area (71%) is used for cultivation on the dairy farm since that provides the best value and gross margin due to dairy production (Table 2B). In addition to grass, cereals and oilseeds for feed are cultivated since they are well suited in crop rotations. The average crop yields are higher on a dairy farm than on the cereal farm also because of more frequent liming (Table 3). Fungicides are used at the same frequency as on cereal farms. The average farm-level gross margin (€211/ha) is almost twice as high as the cereal farm. The average emissions are 3.32 tCO_2_e/ha for all field parcels of the dairy farm and 27.9 tCO_2_e/ha for the organic soil parcels. Perennial grass is dominant on the organic parcels, but also cereals and oilseed are cultivated occasionally. The total production calculated as a feed energy is close to the production of the cereal farm, but the GHG emissions and emissions per produced unit are much lower. Crop production on the dairy farm matches quite well with the feeding requirement for dairy cattle (Huhtamäki 2021).

### Scenario settings

The effects of the GHG emissions tax on land emissions and farm-level production were analyzed by constructing different tax scenarios for a cereals farm (CF) and a dairy farm (DF). The farm-level emissions are based on the farm-level land use and CO_2_e emission estimates for different land-use options estimated by the IPCC (2014) (Table 1 in section 2.3.2).

For each scenario, the emission-tax rates between €0 and 50 €/tCO2e were used. The following costs and subsidies were assumed fixed in all emissions tax scenarios. In an organic field parcel, there was an option to invest in adjustable drainage to reduce greenhouse gas emissions. Investment subsidies currently paid for adjustable drainage in Finland were considered. The minimum accepted cost eligible for a subsidy is €3000 and maximum subsidy is 40% of the total accepted costs (Pro Agria 2019; Finnish Food Authority 2021). Adjustable drainage is subsidized at €70/ha by the CAP agri-environment scheme that is implemented at the national level. A subsidy is provided also for underground watering and runoff water recycling. Subsidies for these amount to €250/ha (ibid, Hyvönen et al., 2020). In this study, it is assumed that the total cost of adjustable drainage investment is €5000/ha and the investment subsidy covers €2000/ha of the costs. From the CAP agri-environment scheme, the farmer receives a €70/ha subsidy annually for maintenance (ibid). Maintenance, monitoring, and adjustment costs are assumed €50/ha annually.

The following scenarios were simulated. **Base scenarios** for both farm types with a 10% organic soil-type area were simulated assuming average prices and crop yields in the region during 2010–2019. The scenarios were run with and without the option to invest in adjustable drainage.

**Sensitivity analyses** were made for three different cases. First, scenarios where controlled drainage results in a *10% and 20% decrease or 10% increase* in crop yields on organic soil parcels were simulated assuming average prices. In the second case, scenarios *20% lower and higher output prices* were simulated. In the third case, it was assumed that *30% of farm area is of organic soil-type*, assuming average prices and no yield effects from controlled drainage investment. This 30% area of organic soils was divided into three different equally sized parcels that cover 30% of the total ten parcels at the farm.

## Results

### Emissions tax with base prices and no yield effect

#### Cereals farm

When an emissions tax is levied and adjustable drainage has no effect on the yields, low levels of emissions tax change the land allocation on the organic soil parcel (Table 4). Considering emissions tax levels €0–15/tCO2e, the higher the emissions tax is, the higher the share of set-aside land on the organic soil parcel is since GHG emissions from set-asides in organic soils are considerably lower than emissions from cereals. The threshold tax level for adjustable drainage investment is €15.1/tCO2e. About 16% reduction is then implied in GHG emissions and a sizable 32% reduction in the net present value (NPV) representing the farm income, while the GHG abatement cost drops from €80/tCO_2_e down to €22/tCO_2_e. After investing in adjustable drainage, production is more intense on the organic soil parcel and thus the share of set-aside land is lower.

**Table 4:** Results for a cereals farm with an adjustable drainage option. Comparison to zero emissions tax results in parenthesis unless otherwise stated. Emissions tax 15.1 €/tCO2eq triggers drainage investment

Emissions tax, €/tCO_2_e	Adjustable drainage decision	% of set-asides on organic soil parcel	Average NPV, €/ha	Average emissions, tCO_2_e/year	Average emissions from organic soil, tCO_2_e/ha parcel (% of total GHG emissions)	Average emissions from mineral soil, tCO_2_e/ha parcel	Cost per of reduced emissions, €/tCO_2_e	Production (TJ/ha)
0	0	0%	110	50.5	35.1 (69.5%)	1.71	0.0	34 681
3	0	10%	103 (−6.4%)	49.6 (−0.9%)	34.1 (68.8%)	1.72	77.3	34 367 (−0.9%)
6	0	17%	96 (−13.3%)	48.9 (−1.6%)	33.5 (68.4%)	1.71	91.8	34 777 (−1.3%)
9	0	23%	90 (−18.7%)	48.4 (−2.1%)	32.8 (67.8%)	1.73	97.5	34 202 (−1.4%)
12	0	35%	83 (−25.1%)	47.2 (−3.3%)	31.7 (67.1%)	1.73	83.8	33 656 (−3.0%)
15	0	43%	76 (−30.7%)	46.3 (−4.2%)	30.9 (66.7%)	1.71	80.3	33 389 (−3.7%)
15.1	1	25%	75 (−31.9%)	34.6 (−15.9%)	19.5 (56.3%)	1.68	22.1	33 138 (−4.5%)
18	1	27%	71 (−35.3%)	34.5 (−16.0%)	19.4 (56.2%)	1.68	24.3	33 087 (−4.6%)
21	1	35%	66 (−39.8%)	33.9 (−16.6%)	18.9 (55.7%)	1.67	26.4	32 895 (−5.1%)
24	1	43%	62 (−44.1%)	33.3 (−17.2%)	18.4 (55.1%)	1.66	28.2	32 224 (−7.1%)
27	1	48%	57 (−48.3%)	32.8 (−17.7%)	18.1 (55.0%)	1.64	30.1	31 786 (−8.3%)
30	1	55%	52 (−53.1%)	32.3 (−18.2%)	17.6 (54.6%)	1.63	32.3	30 780 (−11.2%)
35	1	65%	45 (−59.0%)	31.9 (−18.6%)	17.0 (53.4%)	1.65	35.0	31 426 (−9.4%)
40	1	73%	38 (−65.9%)	31.3 (−19.2%)	16.5 (52.8%)	1.64	37.9	30 900 (−10.9%)
45	1	82%	31 (−71.9%)	30.9 (−19.6%)	16.0 (51.9%)	1.65	40.3	30 758 (−11.3%)
50	1	87%	24 (−78.1%)	30.7 (−19.8%)	15.7 (51.2%)	1.66	43.5	30 717 (−11.4%)

When increasing the emissions tax more than the threshold tax, the area set aside on the organic parcel increases again. The higher the emissions tax, the higher the share of set-aside on organic soil parcel (Table 4).

If there is no option for adjustable drainage investment, the results suggest that the overall GHG emissions would be about 30% higher and emissions from organic soil parcel would be 46–61% higher than in a situation where adjustable drainage investment is available as an option (see Table A2 in Appendix). Without adjustable drainage-investment option, the emissions tax would cut the average gross margins remarkably and the costs of further emission cuts would be high.

Land use on other field parcels than organic soil parcel differs very little between the emissions tax scenarios with and without adjustable drainage investment.

From the farmer point of view, the emissions tax can be economically expensive. When the emissions tax is €21/CO_2_e, emissions decrease by 16.6%, but simultaneously, the average gross margin decreases by 39.8%. The gross margin decreases relatively more than GHG emissions if the emissions tax rate increases further. GHG emissions per hectare are approximately 18–20 times higher for the organic soil parcel than mineral soil parcels when an adjustable drainage investment is not made, and 9.5–12 times higher if there is an adjustable drainage investment. Production counted as feed-energy units decreases by 4.5–11.5%.

With the current input and output prices and subsidy policy, production on the organic soil parcel becomes unprofitable already at low levels of emissions tax. After investing in adjustable drainage and utilizing the maximum set-aside area, further GHG emissions cuttings at high levels of emissions tax are small, and they become costly for a farmer since the farm-level average NPV/ha drops drastically (Table 4).

#### Dairy farm

Unlike the cereals farm, the dairy farm gets value from grass-forage output. When the emissions tax is €0–19/tCO_2_e, the dairy farm allocates more grass to the organic soil parcel to avoid some GHG emissions tax expenses (Table 5). The emissions decrease only slightly as the emissions tax rate increases because the dairy farm produces grass on the organic soil parcel already without the emissions tax. An adjustable drainage investment is profitable when the emissions tax is €19.1/tCO2e. At tax levels €19.1–50/tCO_2_e emissions, are cut by 32–35% and the NPV/ha by 15.5–33%. Slightly above the threshold tax, the share of grass on the organic soil parcel decreases again and cereals are more often produced. The organic soil parcel is put permanently used for grass when the emissions tax is about €30/tCO2e. At an emissions tax level of €21/tCO_2_e, the average gross margin is only 17% lower, but GHG emissions are 31% lower than in the situation where there is no emissions tax. The total production decreases only slightly as the emissions tax rate increases. This is because the value of the grass output is relatively high. On a dairy farm the difference in farm income, NPV/ha, between cases with and without the adjustable drainage at an emissions tax rate of €21–50/tCO2e, is just 0.5–10.4%, whereas the difference in emissions is 34.8–40.5% (see the comparison in Appendix Table A3). Hence, a GHG emissions reductions can be achieved with relatively small income losses.

**Table 5:** Results for a dairy farm with an adjustable drainage option. Comparison to zero emissions tax results in parenthesis unless otherwise stated. Emissions tax 19.1 €/tCO2eq triggers drainage investment

GHG Emissions tax, tCO_2_e	Adjustable drainage decision	Overall grass area of total area	% of grass & NMF at organic soil parcel	Average NPV, €/ha	Average emissions, tCO_2_e/year	Average emissions from organic soil, tCO_2_e/ha parcel (% of total GHG emissions)	Average emissions from mineral soil, tCO_2_e/ha parcel	Cost per of reduced emissions, €/tCO_2_eq	Production (TJ/ha)
0	0	71%	73%	211	39.5	27.9 (70.6%)	1.29		35 136
3	0	72%	76%	205 (−2.6%)	39.2 (−0.8%)	27.7 (70.5%)	1.29	170.0	34 881 (−0.7%)
6	0	73%	81%	201 (−4.9%)	38.7 (−2.1%)	27.2 (70.2%)	1.28	121.9	34 859 (−0.8%)
9	0	75%	87%	195 (−7.6%)	38.0 (−3.8%)	26.6 (70.0%)	1.27	105.3	34 604 (−1.5%)
12	0	75%	89%	190 (−10.0%)	37.7 (−4.5%)	26.4 (69.8%)	1.27	117.4	34 573 (−1.6%)
15	0	75%	90%	185 (−12.5%)	37.7 (−4.7%)	26.3 (69.8%)	1.27	140.3	34 591 (−1.6%)
18	0	76%	98%	180 (−14.9%)	36.8 (−6.8%)	25.5 (69.1%)	1.26	115.9	34 352 (−2.2%)
19	0	77%	100%	178 (−15.6%)	36.6 (−7.4%)	25.3 (69.1%)	1.26	113.2	34 207 (−2.6%)
19.1	1	76%	90%	178 (−15.6%)	26.8 (−32.1%)	15.5 (57.8%)	1.26	26.0	34 500 (−1.8%)
21	1	74%	87%	175 (−17.0%)	27.2 (−31.3%)	15.7 (57.9%)	1.27	29.0	34 605 (−1.5%)
24	1	76%	90%	172 (−18.5%)	26.8 (−32.2%)	15.5 (57.8%)	1.26	30.6	34 438 (−2.0%)
27	1	77%	98%	168 (−20.2%)	26.3 (−33.5%)	15.0 (57.0%)	1.26	32.2	34 143 (−2.8%)
30	1	78%	100%	164 (−22.0%)	26.1 (−34.0%)	14.9 (57.1%)	1.25	34.6	33 999 (−3.2%)
35	1	78%	100%	159 (−24.7%)	26.1 (−33.9%)	14.9 (57.0%)	1.25	38.9	34 144 (−2.8%)
40	1	79%	100%	153 (−27.6%)	26.0 (−34.3%)	14.9 (57.4%)	1.23	42.8	33 767 (−3.9%)
45	1	79%	100%	146 (−30.6%)	26.0 (−34.3%)	14.9 (57.4%)	1.23	47.6	33 786 (−3.8%)
50	1	81%	100%	141 (−33.3%)	25.8 (−34.8%)	14.9 (57.8%)	1.21	51.1	33 530 (−4.6%)

### Sensitivity Analysis

#### Emissions tax with base prices and a 10–20% yield effect

If the adjustable drainage leads to a 10% lower crop yield than in the base scenarios, the emissions tax level to trigger investment is higher (CF €18.8/tCO_2_e, DF €26.0/tCO_2_e) (Appendix Table A4, A7). Up to this emissions tax level, farms adjust their production in a similar way as in the base scenarios when facing an emissions tax by increasing shares of set-asides (CF) or grass (DF). About 20% lower yields would increase the threshold level further (CF €20.1/tCO_2_e, DF €32.9/tCO_2_e) (Appendix Table A5, A8). In the case of cereals, farm production in organic soil parcel would diminish almost completely.

Even a relatively small 10% crop-yield gain (Appendix Table A6, A9) reduces the emissions tax rate needed for triggering adjustable drainage investments for both farm types (in both €11.5/tCO_2_e), compared with the base-scenario cases, assuming no yield gains. This is understandable since the value and gross margin of all crops increase on the organic soil field parcel because of adjustable drainage. The organic soil parcel is more frequently kept in agricultural production—rather than put on set-aside—if adjustable drainage provides yield gains. More intense production leads to higher GHG emissions from the organic soil parcel, but the overall emissions of the farm are also only slightly higher. As a result, the emissions per unit produced calculated as feed-energy content change only a little.

#### Effects of 20% higher or lower crop prices

When considering no yield effect but 20% higher or lower crop prices, the emissions tax rate needed to trigger adjustable drainage investments does not change much compared with the situation for the current output prices (see Appendix Tables A10–A13 for more results of the sensitivity analysis). Changes in the output prices change the gross margins of every soil parcel, not just the organic soil parcel.

#### A 30% share organic soil parcel of the overall land area

Here equally sized parcels 1 (0.5 km from the farm center), 6 (2 km), and 10 (7 km) are of an organic soil type covering 30% of the total farmland area.

The results for the cereals farm can be seen in Table 6. When the emissions tax is low (€0–11.3/tCO_2_e), it is more profitable to put organic soil-type parcels to set-aside (with perennial grass cover) rather than investing in controlled drainage. Setting the parcel aside implies less GHG emissions than the emissions from annual crops such as cereals and thus a lower emissions tax bill. Organic soil-type parcels (1 and 6), which are close to the farm center are allocated as nature-management fields and the furthest parcel (10) is set aside. When the emissions tax is €11.4/tCO_2_e, an investment for adjustable drainage is made for parcel 6. Production on parcel 6 intensifies since more cereals are produced on that parcel, whereas the share of land set aside increases in other organic soil-type parcels 1 and 10. An adjustable drainage investment is made also for parcel 1 when the emissions tax is €11.9/tCO_2_e and for parcel 10 when the tax is €12.3/tCO_2_e. The emissions tax cuts the gross margins significantly and already at emissions tax levels over €27/tCO_2_e, production at the farm becomes unprofitable. Without the possibility for adjustable drainage, the NPV of the cereals farm becomes negative and the production of annual crops decreases drastically on the entire farm if the GHG emissions tax exceeds €22/tCO_2_q.

**Table 6:** Results for a cereals farm which has 30% share of organic soil types of total area. Comparison to zero emissions tax results in parenthesis unless otherwise stated. Emissions tax €11.5–12.3tCO_2_e triggers drainage investments

Emissions tax, €/tCO_2_e	Adjustable drainage decision (parcel 1, parcel 6, parcel 10)	Average NPV, €/ha	Average emissions, tCO_2_e/year	Average emissions from organic soil, tCO_2_e/ha parcel (% of total GHG emissions)	Cost per of reduced emissions, €/tCO_2_e	Production (TJ/ha)
0	0, 0, 0	113	115.3	103.5 (89.7%)	0	34 618
3	0, 0, 0	96 (−14.8%)	114.5 (−0.8%)	102.7 (89.7%)	204.4	34 139
6	0, 0, 0	82 (−27.6%)	112.3 (−3.0%)	100.1 (89.1%)	103.0	34 616
9	0, 0, 0	66 (−41.5%)	109.9 (−5.5%)	97.1 (88.4%)	85.5	34 258
12	1, 1, 0	51 (−54.8%)	83.0 (−32.3%)	70.7 (85.2%)	19.2	33 934
15	1, 1, 1	41 (−63.9%)	70.3 (−45.1%)	57.9 (82.4%)	16.0	33 581
18	1, 1, 1	31 (−73.0%)	69.8 (−45.5%)	57.6 (82.5%)	18.1	32 914
21	1, 1, 1	22 (−80.8%)	68.5 (−46.9%)	55.8 (81.5%)	19.5	33 201
24	1, 1, 1	11 (−90.0%)	67.2 (−48.1%)	54.6 (81.2%)	21.1	31 662
27	1, 1, 1	2 (−97.9%)	67.0 (−48.3%)	54.5 (81.3%)	22.9	31 447

The dairy farm (Table 7) has more room to adjust its production when facing an emissions tax since grass forage produces value added for the farm. The dairy farm increases its overall grass production and especially grass production on organic soil-type parcels in order to avoid emissions tax payments. Adjustable drainage investments are made for all three parcels of organic soil if the emissions tax reaches €18.3–19.1/CO_2_e. The implications of the adjustable drainage on land use and production on the farm are similar, but the NPV drops significantly when 30% of land is of an organic soil type compared with the case when 10% of land is of an organic soil type (Table 5).

**Table 7:** Results for a dairy farm which has 30% share of organic soil types of total area. Comparison to zero emissions tax results in parenthesis unless otherwise stated. Emissions tax €18.3–19.1tCO_2_e triggers drainage investments

Emissions tax, €/tCO_2_e	Adjustable drainage decision (parcel 1, parcel 6, parcel 10)	Overall grass area of total area	Average NPV, €/ha	Average emissions, tCO_2_e/year	Average emissions from organic soil, tCO_2_e/ha parcel (% of total GHG emissions)	Cost per of reduced emissions, €/tCO_2_e	Production (TJ/ha)
0	0, 0, 0	69%	210	94.4	85.3 (90.3%)	0	35 089
3	0, 0, 0	70%	198 (−5.8%)	93.4 (−1.0%)	84.3 (90.2%)	128.9	35 193
6	0, 0, 0	74%	184 (−12.3%)	91.7 (−2.9%)	82.7 (90.2%)	95.7	34 592
9	0, 0, 0	74%	172 (−18.3%)	90.4 (−4.3%)	81.3 (90.0%)	95.3	34 419
12	0, 0, 0	77%	161 (−23.5%)	88.7 (−6.0%)	79.8 (89.9%)	87.8	34 158
15	0, 0, 0	78%	148 (−29.5%)	87.8 (−7.0%)	78.8 (89.8%)	93.9	33 916
18	1, 1, 1	78%	137 (−35.0%)	87.1 (−7.7%)	78.2 (84.2%)	101.2	33 906
21	1, 1, 1	78%	128 (−39.2%)	55.7 (−40.9%)	46.9 (84.2%)	21.3	33 862
24	1, 1, 1	79%	120 (−42.8%)	55.4 (−41.3%)	46.7 (84.2%)	23.1	33 793
27	1, 1, 1	80%	113 (−46.2%)	54.9 (−41.9%)	46.0 (83.9%)	24.6	33 630
30	1, 1, 1	79%	106 (−49.7%)	55.1 (−41.6%)	46.3 (84.0%)	26.6	33 790
35	1, 1, 1	83%	93 (−55.6%)	53.4 (−43.4%)	44.7 (83.7%)	28.5	32 862
40	1, 1, 1	84%	80 (−61.9%)	53.3 (−43.5%)	44.7 (83.8%)	31.7	32 640
45	1, 1, 1	84%	68 (−67.4%)	53.3 (−43.6%)	44.7 (83.9%)	34.5	32 556
50	1, 1, 1	84%	57 (−73.1%)	53.3 (−43.5%)	44.7 (83.8%)	37.4	32 732

## Discussion

The results show that a GHG emissions tax can reduce emissions significantly and at reasonable costs on a farm that has an average or higher share of organic soils. Large reductions of GHG emissions can be achieved on such soils by increasing grasslands on organic field parcels, and especially by investing in adjustable drainage, which reduces the costs of GHG emissions, but still allows cultivation of crops using conventional machinery. However, emissions tax can be an expensive instrument for a farmer. Farm income decreases relatively more than the GHG emissions at increasing rates of emissions tax on cereals farms, while on dairy farms, the farm income decreases relatively less than the GHG emissions. Still, the income losses due to emissions tax are significant also on dairy farms. The cost per tCO_2_e-emission reductions is even higher on dairy farms than on cereal farms. Income losses can be compensated using lump sum payments not coupled to production, while the costs of reducing GHG emissions can be reduced to a level of €22–35/tCO_2_e by making investments in adjustable investment when emissions tax rates are €15–30/tCO_2_e. If there is no option for adjustable drainage, GHG abatement costs are as high as €77–125/tCO_2_e, depending on the farm type, according to the results.

If the GHG emissions tax is higher than €30/tCO_2_e, the cost of cutting GHG emission increases and farms have limited means of reducing the GHG emissions after implementing the adjustable drainage. On the studied cereal farm (10% share of peatlands), the costs of GHG-emission reduction increase from €22/tCO_2_e up to €43.5/tCO_2_e when the emissions tax increases from €15.1tCO_2_e (threshold tax) to €50/tCO_2_e. On a dairy farm (10% share of peatland), the costs of GHG-emission reduction increase from €26/tCO_2_e up to €51/tCO_2_e if the emissions tax increases from €19.1/tCO_2_e (threshold tax) to €50/tCO_2_e.

If the share of peatlands was 30% of the available farmland area on the cereals and dairy farm the GHG reduction costs due to adjusted drainage option are between €16 and 45/tCO_2_e, but the farm income decreases 55–97% at emissions tax rates €12–27/tCO_2_e (CF) and 24–62% at tax rates €15–40/tCO_2_e (DF). Hence, the economic viability of agricultural production is severely reduced already in low levels on emissions tax. However, lump sum compensations to avoid income losses due to the emissions tax may prevent income losses but may not maintain production levels and motivation on farms in the long run with high shares of organic soils.

Our results, specific to farms and GHG emissions factors of peatlands in the Boreal pedoclimatic zone in Finland, cannot be directly compared with other studies on farm-level costs of GHG reduction on peatlands in other areas. Krimly et al. (2016) calculated reduction costs for greenhouse gas emission reduction on typical farm types in southern Germany where the average share on peatland on farms was 19%. The farm types and their size were close to the same as in our study. Krimly et al. (2016) used linear-optimization farm-modeling methods, while dynamic optimization model used in this study has several nonlinear components. According to their results, the conversion of arable peat-soil land into medium-drained intensive grassland leads to high reduction costs up to €92/tCO_2_e, while the reduction costs of rewetting and conversion into wet grassland range from €5/tCO_2_e to €57/tCO_2_e. We did not analyze the option of restoration in this study due to lack of data.

Grossmann and Dietrich (2012) used a water-management model and an econometric approach to calculate GHG emissions abatement cost estimates for cases of changed peatland management in the context of agricultural peatlands in the Elbe river basin in North-East Germany. They reported reduction costs as low as €7–14/tCO_2_e for peatland restoration, while the median-estimated reduction costs for certain peatland-stabilization scenarios were within a range of €10–20/tCO_2_e, which is close to our cost estimates. However, wetland restoration implies raising the water table close to the surface and thus the results are not comparable to our study. Our results show reduction costs as low as €22–44/tCO_2_e even if the watertable is raised to 30 cm from surface only. This is encouraging since the watertable can be temporarily adjusted to lower levels and farmers dependent on peatlands, and often living in remote northern areas with abundant peatlands but with few alternative sources of income, could still use peatlands in conventional production while significantly reducing the GHG emissions. True incentives for GHG reduction on peatlands would also send a signal to farmers to reduce their dependency on peatlands.

GHG emissions reduction costs of raising the water table higher than 30 cm from the surface were not calculated in this study. While Koljonen et al. (2017) report low GHG abatement costs at a broad range of €6–20/tCO_2_e, we considered it too early to evaluate such costs due to inadequate cost estimates of raising the watertable, costs of field work on very wet peatlands, and estimates on the value of the crop output in the context of very wet peatlands in Finland. There are too few studies and experiences to our knowledge.

At first was assumed that average yields on organic soils would remain the same when an adjustable-drainage investment has been made. Recent studies related to adjustable drainage have focused on the GHG emissions reduction potential. In the study by Myllys (2019), average yields on adjustable drainage parcels were close to the average yields on organic soil parcels without adjustable drainage, but the variation in the yields was quite large, depending on the weather conditions. With low precipitation and long drought periods during the growing season, adjustable drainage resulted in a yield increase. We found in our sensitivity analysis that if 10% higher long-term average crop yields could be achieved on peatland parcels using adjustable drainage, then the GHG reduction cost becomes smaller, and a lower emissions tax is needed to trigger investments in adjustable drainage. Thus, if crop-yield gains can be reached, then adjustable drainage is an even more economic option for GHG emissions reduction. As climate change proceeds, extreme weather conditions, including long drought periods, will become more frequent, and adjustable drainage might potentially secure more stable yields simultaneously with GHG emissions reduction.

Climate change is prolonging growing seasons in the north and is moving agroclimate zones northward (Ruosteenoja et al. 2011; Rötter et al. 2013; Tao et al. 2015), which might lead to increasing shares of annual crops in production (Purola et al. 2018). This would lead to increasing GHG emissions from the organic soils. Our results suggest that adjustable drainage and emissions taxes can contribute to keeping the GHG emissions low or reducing them.

Current policy includes voluntary measures such as payments for permanent grasslands on peatlands. This has not been popular among farmers. Our results suggest that grasslands on peatlands do not facilitate low-cost GHG emissions reductions on cereal farms or on dairy farms, but grasslands can still contribute to reasonably priced GHG emissions reductions if coupled to adjustable drainage with increased watertable levels. Thus, incentivizing adjustable drainage should be emphasized in climate policy aiming for cost-effective GHG emissions reduction on peatlands, especially on farms, which cannot operate on very wet peatlands.

## Conclusions

There is increasing demand to reduce GHG emissions in agriculture. Significant reductions of GHG emissions can be achieved on agricultural peatlands by using adjustable drainage systems and by increasing grassland cultivation. However, there has been little incentive for farmers to reduce GHG emissions on peatlands in Finland and most of the EU.

Our results based on dynamic farm modeling over a period of 30 years applied to a cereal and dairy-farm case in southwest Finland show that emissiosn tax can be an effective policy instrument if adjustable drainage is available as an option. Tax rates of €12–20/tCO_2_e are sufficient, depending on the farm type and share of peatlands (10–30% in our analysis) to trigger adjustable drainage on peatlands and imply significant GHG emissions reductions on both cereals and dairy farms. The cost of reducing GHG emissions is approximately €16–26/tCO_2_e by making an adjustable-drainage investment in combination with grassland management in our calculated cases. These GHG abatement costs are not much higher than those calculated for cases of establishing very wet peatlands, reported in other European studies.

Our results also show that higher emissions tax than €20/tCO_2_e lead to high reduction costs. On a cereal farm (share of peatlands 10%), the cost of GHG emissions reduction increases from €22/tCO_2_e up to €43.5/tCO_2_e if the emissions tax is increased from €15/tCO_2_e to €50/tCO_2_e. On a dairy farm (share of peatlands 10%), the cost of GHG emissions reduction increases from €26/tCO_2_e up to €51/tCO_2_e when the emissions tax increases from €19/tCO_2_e to €50/tCO_2_e. This is because farms have limited means of reducing the GHG emissions further after implementing the adjustable drainage.

An emissionst tax can be an expensive instrument for farmers, even if applying the adjustable drainage option, due to income losses 15–55% in our Finnish farm cases, if not compensated by lumpsum payments not coupled to the production of the farm, or to the land. Introducing such a payment would not destroy the incentive effect of the emissions tax, but major income losses resulting from the emissions tax and implied production and land-use adjustments could be avoided.

The calculated costs of GHG emissions reduction can be considered reasonable when compared with the emission trading prices in the EU, which were higher than €20/tCO_2_e during most of the year in 2020 and reached €50/tCO_2_e in May–June 2021 (EMBER 2021).

If any crop-yield gains can be reached by the means of an adjustable water-table, e.g., during dry-growing periods, then adjustable drainage is little more economic from the farm-level point of view. Small yield gains, however, do not make an adjustable-drainage option economically viable. It is also noteworthy that according to our results, emissions tax is inefficient and expensive in reducing GHG emissions from organic soils if there is no adjustment-drainage option.

Since the current agrienvironmental scheme in Finland is weak in incentivizing GHG emissions reduction on peatlands, based on our results, we see that an emissions tax is needed and would be effective at incentivizing farmers to make GHG emissions reductions on peatlands, while the economic losses to farms should be compensated for by a lumpsum payment fully decoupled from production or land. This opportunity should not be missed since significant GHG emissions reductions are possible on peatlands, covering 11% of agricultural lands in Finland, while the climate targets of Finland are more ambitious than those of the EU. However, practical implementation of emissions tax may be challenging since the soil type of every field parcel may not be possible without ambiguity.

This study contributes also in terms of methodology since we added the adjustable drainage option and GHG emissions tax in a dynamic-optimization model with explicit field parcels and farm-level management with crop rotations and input-use yield responses over a 30-year time span, which is an appropriate length in a climate change-related analysis where long-term investments are also included. We may include the restoration option, i.e., very wet peatlands, not yet included in this study, in further studies of GHG emissions reduction and farm economy.

## Supplementary Information

Supplementary Information

## Data Availability

The data used in this study will be made available upon request.

## Code availability

The code for the dynamic optimization farm models will be made available by authors upon request.
