# Correction to: Farm-Level Effects of Emissions Tax and Adjustable Drainage on Peatlands

**DOI:** 10.1007/s00267-022-01641-8

**Published:** 2022-04-21

**Authors:** Tuomo Purola, Heikki Lehtonen

**Affiliations:** Luke Natural Resources Institute Finland - Economics and society, Latokartanonkaari 9, FI-00790 Helsinki, Finland

Correction to: *Environmental Management* (2021) 69:154–168

10.1007/s00267-021-01543-1 published online 14 October 2021

In table 4, some rows under the column “Average emissions, t/co2e/year”, the minus symbol was placed before percentage instead the minus symbol should have been placed before numbers. The Table 4 has now been corrected.

The original article has been corrected.

