# Supplementary material for: Farm-Level Effects of Emissions Tax and Adjustable Drainage on Peatlands
Source: Environ Manage. 2021 Oct 14;69(1):154–68. doi: 10.1007/s00267-021-01543-1 (PMC8758597; doi:10.1007/s00267-021-01543-1)
Supplement: Supplementary file 1 — Supplementary Information [file 267_2021_1543_MOESM1_ESM.docx]

## Appendix. Input data and additional farm modelling results in selected cases

Table A1. Average crop yields, crop prices, variable costs of production and farm subsidies in Varsinais-Suomi region. Sources: Official Farm Statistics (OFS) (2020), DREMFIA sector model (Lehtonen and Niemi, 2018).

| Land use option | Average yield, kg/ha | Output price, €/kg | Variable costs, €/ha | Subsidies, €/ha |
| --- | --- | --- | --- | --- |
| Spring wheat | 3936 | 0.1785 | 609 | 539 |
| Winter wheat | 4359 | 0.1785 | 669 | 578 |
| Feed barley | 3915 | 0.1483 | 561 | 579 |
| Malting barley | 3899 | 0.174 | 617 | 539 |
| Oats | 3884 | 0.1515 | 580 | 579 |
| Oilseed rape | 1668 | 0.1837 | 636 | 627 |
| Grass | 13 595 | 0.3618 | 760 | 579 |
| Set-aside | - | 0.0653 | 301 | 407 |
| NMF | - | - | 321 | 467 |

Table A2. Results for a **cereals** farm **without** an adjustable drainage option Comparison to results with adjustable drainage investment in parenthesis unless otherwise stated.

| Emissions tax, €/tCO2e | % of set-asides on organic soil parcel | Average NPV, €/ha | Average emissions, tCO2e/year | Average emissions from organic soil, tCO2e/ha parcel | Cost per  of reduced emissions, €/tCO2e |
| --- | --- | --- | --- | --- | --- |
| 18 | 53%(27%) | 70(-1.9%) | 45.3(+31.5%) | 29.9(+54.2%) | 78.3(24.3) |
| 21 | 70%(35%) | 64(-3.7%) | 43.7(+28.8%) | 28.2(+49.7%) | 67.8(26.4) |
| 24 | 82%(43%) | 58(-5.9%) | 42.5(+27.7%) | 27.1(+47.6%) | 65.3(28.2) |
| 27 | 88%(48%) | 52(-9.0%) | 41.9(+27.6%) | 26.4(+46.5%) | 67.7(30.1) |
| 30 | 93%(55%) | 47(-10.0%) | 41.4(+27.9%) | 26.0(+47.1%) | 69.8(32.3) |
| 35 | 100%(65%) | 37(-19.0%) | 40.7(+27.6%) | 25.3(+48.5%) | 75.1(35.0) |
| 40 | 100%(73%) | 27(-27.7%) | 40.6(+29.5%) | 25.3(+53.1%) | 83.7(37.9) |
| 45 | 100%(82%) | 18(-42.8%) | 40.5(+31.2%) | 25.3(+57.9%) | 92.2(40.3) |
| 50 | 100%(87%) | 9(-63.5%) | 40.5(+32.0%) | 25.3(+61.0%) | 101.5(43.4) |

Table A3. Results for a **dairy** farm **without** an adjustable drainage option. Comparison to results with an adjustable drainage investment in parenthesis unless otherwise stated.

| GHG emissions tax, tCO2e | Overall grass area of total area | Share of grass and NMF at organic soil parcel | NPV €/ha/year | Average emissions, tCO2e/year | Average emissions from organic soil, tCO2e/ha parcel | Cost per ton  of reduced emissions |
| --- | --- | --- | --- | --- | --- | --- |
| 21 | 77%(%) | 100%(87%) | 174(-0.5%) | 36.6(+34.8%) | 25.3(+61.0%) | 124.8(29.0) |
| 24 | 77%(%) | 100%(90%) | 170(-1.4%) | 36.6(+36.6%) | 25.3(+63.1%) | 141.5(30.6) |
| 27 | 77%(%) | 100%(98%) | 164(-2.4%) | 36.6(+39.0%) | 25.3(+68.6%) | 156.2(32.2) |
| 30 | 78%(%) | 100%(100%) | 159(-3.1%) | 36.5(+39.7%) | 25.3(+69.8%) | 168.0(34.6) |
| 35 | 78%(%) | 100%(100%) | 151(-4.7%) | 36.5(+39.9%) | 25.3(+69.8%) | 199.2(38.9) |
| 40 | 79%(%) | 100%(100%) | 143(-6.6%) | 36.4(+40.1%) | 25.3(+69.8%) | 216.2(42.8) |
| 45 | 80%(%) | 100%(100%) | 134(-8.5%) | 36.3(+39.7%) | 25.3(+69.8%) | 236.8(47.6) |
| 50 | 81%(%) | 100%(100%) | 126(-10.4%) | 36.2(+40.5%) | 25.3(+69.8%) | 257.3(51.1) |

## Table A4. 10% yield decrease in organic soil parcel with base prices in a cereals farm. Base scenario results without yield decrease in parenthesis. Emissions tax €18.8/tCO2e triggers drainage investment (€15.1/tCO2e if no yield increase).

| Emissions tax, €/tCO2e | Adjustable drainage decision | % of set-aside on organic soil parcel | Average NPV, €/ha | Average emissions, tCO2e/year | Cost  of reduced emissions, €/tCO2e |
| --- | --- | --- | --- | --- | --- |
| 0 | 0(0) | 0%(0%) | 110(110) | 50.5(50.5) | - |
| 3 | 0(0) | 10%(10%) | 103(103) | 49.6(49.6) | 77.3(77.3) |
| 6 | 0(0) | 17%(17%) | 96(96) | 48.9(48.9) | 91.8(91.8) |
| 9 | 0(0) | 23%(23%) | 90(90) | 48.4(48.4) | 97.5(97.5) |
| 12 | 0(0) | 35%(35%) | 83(83) | 47.2(47.2) | 83.8(83.8) |
| 15 | 0(0) | 43%(43%) | 76(76) | 46.3(46.3) | 80.3(80.3) |
| 18 | 0(1) | 57%(27%) | 70(71) | 44.9(34.5) | 72.2(24.3) |
| 21 | 1(1) | 60%(35%) | 65(66) | 32.7(33.9) | 25.5(26.4) |
| 24 | 1(1) | 63%(43%) | 60(62) | 32.1(33.3) | 27.4(28.2) |
| 27 | 1(1) | 73%(48%) | 56(57) | 31.6(32.8) | 28.9(30.1) |
| 30 | 1(1) | 80%(55%) | 52(52) | 31.3(32.3) | 30.4(32.3) |
| 35 | 1(1) | 88%(65%) | 44(45) | 30.8(31.9) | 33.5(35.0) |
| 40 | 1(1) | 93%(73%) | 38(38) | 30.5(31.3) | 36.2(37.9) |
| 45 | 1(1) | 100%(82%) | 31(31) | 30.1(30.9) | 39.1(40.3) |
| 50 | 1(1) | 100%(87%) | 23(24) | 30.0(30.7) | 42.7(43.5) |

## Table A5. 20% yield decrease in organic soil parcel with base prices in a cereals farm. Base scenario results without yield decrease in parenthesis. Emissions tax €20.1/tCO2e triggers drainage investment (€15.1/tCO2e if no yield increase).

| Emissions tax, €/tCO2e | Adjustable drainage decision | % of set-aside on organic soil parcel | Average NPV, €/ha | Average emissions, tCO2e/year | Cost  of reduced emissions, €/tCO2e |
| --- | --- | --- | --- | --- | --- |
| 0 | 0(0) | 0%(0%) | 110(110) | 50.5(50.5) | - |
| 3 | 0(0) | 10%(10%) | 103(103) | 49.6(49.6) | 77.3(77.3) |
| 6 | 0(0) | 17%(17%) | 96(96) | 48.9(48.9) | 91.8(91.8) |
| 9 | 0(0) | 23%(23%) | 90(90) | 48.4(48.4) | 97.5(97.5) |
| 12 | 0(0) | 35%(35%) | 83(83) | 47.2(47.2) | 83.8(83.8) |
| 15 | 0(0) | 43%(43%) | 76(76) | 46.3(46.3) | 80.3(80.3) |
| 18 | 0(1) | 57%(27%) | 70(71) | 44.9(34.5) | 72.2(24.3) |
| 21 | 1(1) | 90%(35%) | 64(66) | 31.2(33.9) | 23.7(26.4) |
| 24 | 1(1) | 95%(43%) | 61(62) | 30.8(33.3) | 25.2(28.2) |
| 27 | 1(1) | 100%(48%) | 55(57) | 30.6(32.8) | 27.5(30.1) |
| 30 | 1(1) | 100%(55%) | 51(52) | 30.4(32.3) | 29.2(32.3) |
| 35 | 1(1) | 100%(65%) | 44(45) | 30.3(31.9) | 32.7(35.0) |
| 40 | 1(1) | 100%(73%) | 38(38) | 30.2(31.3) | 35.7(37.9) |
| 45 | 1(1) | 100%(82%) | 30(31) | 30.2(30.9) | 39.4(40.3) |
| 50 | 1(1) | 100%(87%) | 24(24) | 30.1(30.7) | 42.5(43.5) |

## Table A6. 10% yield increase in organic soil parcel with base prices in a cereals farm. Base scenario results without yield increase in parenthesis. Emissions tax €11.6/tCO2e triggers drainage investment (€15.1/tCO2e if no yield increase).

| Emissions tax, €/tCO2e | Adjustable drainage decision | % of set-aside on organic soil parcel | Average NPV, €/ha | Average emissions, tCO2e/year | Cost  of reduced emissions, €/tCO2e |
| --- | --- | --- | --- | --- | --- |
| 0 | 0(0) | 0%(0%) | 110(110) | 50.5(50.5) | - |
| 3 | 0(0) | 10%(10%) | 103(103) | 49.6(49.6) | 77.3(77.3) |
| 6 | 0(0) | 17%(17%) | 96(69) | 48.9(48.9) | 91.8(91.8) |
| 9 | 0(0) | 23%(23%) | 90(90) | 48.4(48.4) | 97.5(97.5) |
| 12 | 1(0) | 0%(35%) | 84(83) | 35.9(47.2) | 18.0(83.8) |
| 15 | 1(0) | 5%(43%) | 79(76) | 35.7(46.3) | 20.9(80.3) |
| 18 | 1(1) | 8%(27%) | 74(71) | 35.3(34.5) | 23.7(24.3) |
| 21 | 1(1) | 12%(35%) | 68(66) | 34.9(33.9) | 27.2(26.4) |
| 24 | 1(1) | 20%(43%) | 64(62) | 34.4(33.3) | 29.0(28.2) |
| 27 | 1(1) | 23%(48%) | 59(57) | 33.9(32.8) | 31.0(30.1) |
| 30 | 1(1) | 28%(55%) | 55(52) | 33.8(32.3) | 33.2(32.3) |
| 35 | 1(1) | 38%(65%) | 47(45) | 33.0(31.9) | 36.3(35.0) |
| 40 | 1(1) | 42%(73%) | 39(38) | 32.7(31.3) | 39.7(37.9) |
| 45 | 1(1) | 47%(82%) | 31(31) | 32.4(30.9) | 43.8(40.3) |
| 50 | 1(1) | 62%(87%) | 24(24) | 31.7(30.7) | 46.1(43.5) |

## Table A7. 10% yield decrease in organic soil parcel with base prices in a dairy farm. Base scenario results without yield decrease in parenthesis. Emissions tax €26.0/tCO2e triggers drainage investment (€19.1/tCO2e if no yield increase).

| Emissions tax, €/tCO2e | Adjustabledrainage decision | Overall grass area of total area | Grass & NMF in organic soil parcel | Average NPV, €/ha | Average emissions, tCO2e/year | Cost  of reduced emissions, €/tCO2e |
| --- | --- | --- | --- | --- | --- | --- |
| 0 | 0(0) | 71%(71%) | 73%(73%) | 211(211) | 39.5(39.5) | - |
| 3 | 0(0) | 72%(72%) | 76%(76%) | 205(205) | 39.2(39.2) | 170.0(170.0) |
| 6 | 0(0) | 73%(73%) | 81%(81%) | 201(201) | 38.7(38.7) | 121.9(121.9) |
| 9 | 0(0) | 75%(75%) | 87%(87%) | 195(195) | 38.0(38.0) | 105.3(105.3) |
| 12 | 0(0) | 75%(75%) | 89%(89%) | 190(190) | 37.7(37.7) | 117.4(117.4) |
| 15 | 0(0) | 75%(75%) | 90%(90%) | 185(185) | 37.7(37.7) | 140.3(140.3) |
| 18 | 0(0) | 76%(76%) | 98%(98%) | 180(180) | 36.8(36.8) | 115.9(115.9) |
| 21 | 0(1) | 76%(74%) | 100%(87%) | 174(175) | 36.7(27.2) | 127.2(29.0) |
| 24 | 0(1) | 76%(76%) | 100%(90%) | 169(172) | 36.7(26.8) | 145.0(30.6) |
| 27 | 1(1) | 78%(77%) | 100%(98%) | 165(168) | 26.1(26.3) | 34.3(32.2) |
| 30 | 1(1) | 78%(78%) | 100%(100%) | 161(164) | 26.1(26.1) | 36.9(34.6) |
| 35 | 1(1) | 78%(78%) | 100%(100%) | 155(159) | 26.1(26.1) | 41.2(38.9) |
| 40 | 1(1) | 78%(79%) | 100%(100%) | 149(153) | 26.1(26.0) | 45.6(42.8) |
| 45 | 1(1) | 80%(79%) | 100%(100%) | 143(146) | 25.9(26.0) | 49.6(47.6) |
| 50 | 1(1) | 81%(81%) | 100%(100%) | 138(141) | 25.8(25.8) | 53.5(51.1) |

## Table A8. 20% yield decrease in organic soil parcel with base prices in a dairy farm. Base scenario results without yield decrease in parenthesis. Emissions tax 32.9 €/tCO2e triggers drainage investment (€19.1/tCO2e if no yield increase).

| Emissions tax, €/tCO2e | Adjustabledrainage decision | Overall grass area of total area | Grass & NMF in organic soil parcel | Average NPV, €/ha | Average emissions, tCO2e/year | Cost  of reduced emissions, €/tCO2e |
| --- | --- | --- | --- | --- | --- | --- |
| 0 | 0(0) | 71%(71%) | 73%(73%) | 211(211) | 39.5(39.5) | - |
| 3 | 0(0) | 72%(72%) | 76%(76%) | 205(205) | 39.2(39.2) | 170.0(170.0) |
| 6 | 0(0) | 73%(73%) | 81%(81%) | 201(201) | 38.7(38.7) | 121.9(121.9) |
| 9 | 0(0) | 75%(75%) | 87%(87%) | 195(195) | 38.0(38.0) | 105.3(105.3) |
| 12 | 0(0) | 75%(75%) | 89%(89%) | 190(190) | 37.7(37.7) | 117.4(117.4) |
| 15 | 0(0) | 75%(75%) | 90%(90%) | 185(185) | 37.7(37.7) | 140.3(140.3) |
| 18 | 0(0) | 76%(76%) | 98%(98%) | 180(180) | 36.8(36.8) | 115.9(115.9) |
| 21 | 0(1) | 76%(74%) | 100%(87%) | 174(175) | 36.7(27.2) | 127.2(29.0) |
| 24 | 0(1) | 76%(76%) | 100%(90%) | 169(172) | 36.7(26.8) | 145.0(30.6) |
| 27 | 1(1) | 78%(77%) | 100%(98%) | 165(168) | 26.1(26.3) | 34.3(32.2) |
| 30 | 0(1) | 78%(78%) | 100%(100%) | 161(164) | 26.1(26.1) | 36.9(34.6) |
| 35 | 1(1) | 78%(78%) | 100%(100%) | 152(159) | 26.1(26.1) | 43.7(38.9) |
| 40 | 1(1) | 79%(79%) | 100%(100%) | 145(153) | 26.0(26.0) | 48.2(42.8) |
| 45 | 1(1) | 80%(79%) | 100%(100%) | 140(146) | 25.9(26.0) | 51.7(47.6) |
| 50 | 1(1) | 79%(81%) | 100%(100%) | 134(141) | 26.0(25.8) | 56.8(51.1) |

## Table A9. 10% yield increase in organic soil parcel with base prices in a dairy farm. Base scenario results without yield increase in parenthesis. Emissions tax €11.5/tCO2e triggers drainage investment (€19.1/tCO2e if no yield increase).

| Emissions tax, €/tCO2e | Adjustabledrainage decision | Overall grass area of total area | Grass & NMF in organic soil parcel | Average NPV, €/ha | Average emissions, tCO2e/year | Cost  of reduced emissions, €/tCO2e |
| --- | --- | --- | --- | --- | --- | --- |
| 0 | 0(0) | 71%(71%) | 73%(73%) | 211(211) | 39.5(39.5) | - |
| 3 | 0(0) | 72%(72%) | 76%(76%) | 205(205) | 39.2(39.2) | 170.0(170.0) |
| 6 | 0(0) | 73%(73%) | 81%(81%) | 201(201) | 38.7(38.7) | 121.9(121.9) |
| 9 | 0(0) | 75%(75%) | 87%(87%) | 195(195) | 38.0(38.0) | 105.3(105.3) |
| 12 | 1(0) | 73%(75%) | 83%(89%) | 190(190) | 27.4(37.7) | 17.3(117.4) |
| 15 | 1(0) | 73%(75%) | 85%(90%) | 186(185) | 27.3(37.7) | 19.9(140.3) |
| 18 | 1(0) | 75%(76%) | 90%(98%) | 183(180) | 26.9(36.8) | 22.3(115.9) |
| 21 | 1(1) | 77%(74%) | 90%(87%) | 179(175) | 26.8(27.2) | 25.1(29.0) |
| 24 | 1(1) | 76%(76%) | 90%(90%) | 175(172) | 26.8(26.8) | 27.9(30.6) |
| 27 | 1(1) | 76%(77%) | 90%(98%) | 172(168) | 26.8(26.3) | 30.9(32.2) |
| 30 | 1(1) | 77%(78%) | 100%(100%) | 168(164) | 26.2(26.1) | 32.0(34.6) |
| 35 | 1(1) | 78%(78%) | 100%(100%) | 162(159) | 26.1(26.1) | 36.4(38.9) |
| 40 | 1(1) | 79%(79%) | 100%(100%) | 156(153) | 26.0(26.0) | 40.6(42.8) |
| 45 | 1(1) | 79%(79%) | 100%(100%) | 150(146) | 26.0(26.0) | 44.7(47.6) |
| 50 | 1(1) | 79%(81%) | 100%(100%) | 144(141) | 26.0(25.8) | 49.2(51.1) |

Table A10. Results for a **cereals** farm with a controlled drainage option with 20% output price **increase**. Base price results in parenthesis. Emission tax €14.6/tCO2e triggers drainage investment (€15.1/tCO2e with average prices).

| Emissions tax, €/tCO2e | Adjustable drainage decision | % of set-aside on organic soil parcel | Average NPV, €/ha | Average emissions, tCO2e/year | Cost  of reduced emissions, €/tCO2e | Production (TJ/ha) |
| --- | --- | --- | --- | --- | --- | --- |
| 0 | 0(0) | 0%(0%) | 171(110) | 52.0(50.5) |  | 41 577(34 681) |
| 3 | 0(0) | 2%(10%) | 164(103) | 51.9(49.6) | 776.9(77.3) | 41 383(34 367) |
| 6 | 0(0) | 7%(17%) | 157(96) | 51.5(48.9) | 258.4(91.8) | 41 176(33 777) |
| 9 | 0(0) | 17%(23%) | 150(90) | 50.6(48.4) | 146.1(97.5) | 41 005(34 202) |
| 12 | 0(0) | 22%(35%) | 143(83) | 50.0(47.2) | 140.9(83.8) | 40 852(33 656) |
| 15 | 1(0) | 13%(43%) | 136(76) | 37.0(46.3) | 23.2(80.3) | 40 879(33 389) |
| 18 | 1(1) | 22%(27%) | 132(71) | 36.6(34.5) | 25.6(24.3) | 41 102(33 087) |
| 21 | 1(1) | 17%(35%) | 126(66) | 36.6(33.9) | 29.3(26.4) | 40 621(32 895) |
| 24 | 1(1) | 27%(43%) | 121(62) | 36.1(33.3) | 31.7(28.2) | 40 547(32 224) |
| 27 | 1(1) | 28%(48%) | 116(57) | 35.8(32.8) | 34.2(30.1) | 40 019(31 786) |
| 30 | 1(1) | 30%(55%) | 111(52) | 35.7(32.3) | 36.9(32.3) | 39 863(30 780) |
| 35 | 1(1) | 43%(65%) | 103(45) | 34.8(31.9) | 39.8(35.0) | 39 253(31 426) |
| 40 | 1(1) | 45%(73%) | 94(38) | 34.6(31.3) | 44.2(37.9) | 38 965(30 900) |
| 45 | 1(1) | 50%(82%) | 87(31) | 34.3(30.9) | 47.9(40.3) | 38 866(30 758) |
| 50 | 1(1) | 57%(87%) | 78(24) | 33.9(30.7) | 51.2(43.5) | 38 475(30 717) |

Table A11. Results for a **cereals** farm with a controlled drainage option with 20% output price **decrease**. Base price results in parenthesis. Emission tax €16.6/tCO2e triggers drainage investment (€15.1/tCO2e with average prices).

| Emissions tax, €/tCO2e | Adjustable drainage decision | % of set-aside on organic soil parcel | Average NPV, €/ha | Average emissions, tCO2e/year | Cost  of reduced emissions, €/tCO2e | Production (TJ/ha) |
| --- | --- | --- | --- | --- | --- | --- |
| 0 | 0(0) | 7%(0%) | 64(110) | 47.8(50.5) |  | 27 886(34 681) |
| 3 | 0(0) | 10%(10%) | 58(103) | 47.5(49.6) | 233.5(77.3) | 27 882(34 367) |
| 9 | 0(0) | 38%(23%) | 45(90) | 45.2(48.4) | 75.7(97.5) | 27 674(34 202) |
| 12 | 0(0) | 52%(35%) | 39(83) | 44.1(47.2) | 69.0(83.8) | 27 636(33 656) |
| 15 | 0(0) | 62%(43%) | 33(76) | 43.4(46.3) | 70.2(80.3) | 27 434(33 389) |
| 18 | 1(1) | 42%(27%) | 28(71) | 32.3(34.5) | 23.6(24.3) | 27 365(33 087) |
| 21 | 1(1) | 48%(35%) | 24(66) | 32.1(33.9) | 25.9(26.4) | 27 517(32 895) |
| 24 | 1(1) | 52%(43%) | 19(62) | 31.9(33.3) | 28.3(28.2) | 27 433(32 224) |
| 27 | 1(1) | 57%(48%) | 15(57) | 31.7(32.8) | 30.9(30.1) | 27 418(31 786) |
| 30 | 1(1) | 62%(55%) | 10(52) | 31.5(32.3) | 33.0(32.3) | 27 312(30 780) |
| 35 | 1(1) | 70%(65%) | 3(45) | 31.1(31.9) | 36.6(35.0) | 27 338(31 426) |
| 40 | 1(1) | 83%(73%) | -4(38) | 30.6(31.3) | 39.5(37.9) | 27 355(30 900) |
| 45 | 1(1) | 95%(82%) | -11(31) | 30.1(30.9) | 42.5(40.3) | 27 165(30 758) |
| 50 | 1(1) | 100%(87%) | -17(24) | 29.9(30.7) | 45.7(43.5) | 27 027(30 717) |

Table A12. Results for a **dairy** farm with a controlled drainage option with 20% output price **increase.** Base price results in parenthesis. Emissions tax €18.3/tCO2e triggers drainage investment (€19.1/tCO2e with average prices).

| Emissions tax, €/tCO2e | Adjustabledrainage decision | Overall grass area of total area | Grass & NMF in organic soil parcel | Average NPV, €/ha | Average emissions, tCO2e/year | Cost  of reduced emissions, €/tCO2e | Production (TJ/ha) |
| --- | --- | --- | --- | --- | --- | --- | --- |
| 0 | 0(0) | 69%(71%) | 71%(73%) | 281(211) | 40.0(39.5) |  | 36 515(35 136) |
| 3 | 0(0) | 71%(72%) | 70%(76%) | 276(205) | 39.9(39.2) | 611.7(170.0) | 36 305(34 881) |
| 6 | 0(0) | 73%(73%) | 77%(81%) | 270(201) | 39.1(38.7) | 126.8(121.9) | 35 901(34 859) |
| 9 | 0(0) | 73%(75%) | 83%(87%) | 265(195) | 38.5(38.0) | 111.0(105.3) | 35 874(34 604) |
| 12 | 0(0) | 73%(75%) | 87%(89%) | 259(190) | 38.2(37.7) | 125.4(117.4) | 35 898(34 573) |
| 15 | 0(0) | 74%(75%) | 90%(90%) | 255(185) | 37.8(37.7) | 123.2(140.3) | 35 785(34 591) |
| 18 | 0(0) | 74%(76%) | 90%(98%) | 249(180) | 37.8(36.8) | 146.9(115.9) | 35 633(34 352) |
| 21 | 1(1) | 74%(74%) | 90%(87%) | 245(175) | 27.0(27.2) | 27.8(29.0) | 35 443(34 605) |
| 24 | 1(1) | 74%(76%) | 90%(90%) | 242(172) | 27.0(26.8) | 30.6(30.6) | 35 636(34 438) |
| 27 | 1(1) | 76%(77%) | 90%(98%) | 238(168) | 26.8(26.3) | 32.9(32.2) | 35 413(34 143) |
| 30 | 1(1) | 75%(78%) | 90%(100%) | 234(164) | 26.9(26.1) | 35.9(34.6) | 35 380(33 999) |
| 35 | 1(1) | 76%(78%) | 97%(100%) | 228(159) | 26.5(26.1) | 39.5(38.9) | 35 314(34 144) |
| 40 | 1(1) | 78%(79%) | 100%(100%) | 222(153) | 26.1(26.0) | 43.1(42.8) | 34 939(33 767) |
| 45 | 1(1) | 78%(79%) | 100%(100%) | 216(146) | 26.1(26.0) | 46.9(47.6) | 34 807(33 786) |
| 50 | 1(1) | 80%(81%) | 100%(100%) | 210(141) | 25.9(25.8) | 50.7(51.1) | 34 386(33 530) |

Table A13. Results for a **dairy** farm with a controlled drainage option with 20% output price **decrease**. Base price results in parenthesis. Emissions tax €19.1/tCO2e triggers drainage investment (€19.1/tCO2e with average prices).

| Emissions tax, €/tCO2e | Adjustabledrainage decision | Overall grass area of total area | Grass & NMF in organic soil parcel | Average NPV, €/ha | Average emissions, tCO2e/year | Cost  of reduced emissions, €/tCO2e | Production (TJ/ha) |
| --- | --- | --- | --- | --- | --- | --- | --- |
| 0 | 0(0) | 70%(71%) | 69%(73%) | 143(211) | 40.0(39.5) |  | 34 282(35 136) |
| 3 | 0(0) | 71%(72%) | 77%(76%) | 138(205) | 39.3(39.2) | 70.8(170.0) | 34 336(34 881) |
| 6 | 0(0) | 73%(73%) | 83%(81%) | 132(201) | 38.5(38.7) | 70.5(121.9) | 34 025(34 859) |
| 9 | 0(0) | 73%(75%) | 89%(87%) | 127(195) | 38.0(38.0) | 77.9(105.3) | 33 990(34 604) |
| 12 | 0(0) | 74%(75%) | 90%(89%) | 122(190) | 37.8(37.7) | 95.2(117.4) | 33 872(34 573) |
| 15 | 0(0) | 75%(75%) | 100%(90%) | 117(185) | 36.8(37.7) | 81.3(140.3) | 33 651(34 591) |
| 18 | 0(0) | 76%(76%) | 100%(98%) | 112(180) | 36.7(36.8) | 92.8(115.9) | 33 463(34 352) |
| 21 | 1(1) | 74%(74%) | 90%(87%) | 108(175) | 27.0(27.2) | 26.9(29.0) | 33 895(34 605) |
| 24 | 1(1) | 75%(76%) | 100%(90%) | 104(172) | 26.4(26.8) | 28.4(30.6) | 33 628(34 438) |
| 27 | 1(1) | 77%(77%) | 100%(98%) | 100(168) | 26.2(26.3) | 30.9(32.2) | 33 321(34 143) |
| 30 | 1(1) | 76%(78%) | 100%(100%) | 97(164) | 26.3(26.1) | 33.5(34.6) | 33 357(33 999) |
| 35 | 1(1) | 78%(78%) | 100%(100%) | 91(159) | 26.1(26.1) | 37.4(38.9) | 33 240(34 144) |
| 40 | 1(1) | 79%(79%) | 100%(100%) | 85(153) | 26.0(26.0) | 41.4(42.8) | 33 042(33 767) |
| 45 | 1(1) | 79%(79%) | 100%(100%) | 79(146) | 26.0(26.0) | 45.6(47.6) | 33 141(33 786) |
| 50 | 1(1) | 80%(81%) | 100%(100%) | 73(141) | 25.8(25.8) | 49.2(51.1) | 32 768(33 530) |
